# Protein Folding Mechanism of the Dimeric AmphiphysinII/Bin1 N-BAR Domain

**DOI:** 10.1371/journal.pone.0136922

**Published:** 2015-09-14

**Authors:** Tobias Gruber, Jochen Balbach

**Affiliations:** Martin-Luther University Halle-Wittenberg, Institute of Physics, Betty-Heimann Str. 7, 06120, Halle, Germany; University of Leeds, UNITED KINGDOM

## Abstract

The human AmphyphisinII/Bin1 N-BAR domain belongs to the BAR domain superfamily, whose members sense and generate membrane curvatures. The N-BAR domain is a 57 kDa homodimeric protein comprising a six helix bundle. Here we report the protein folding mechanism of this protein as a representative of this protein superfamily. The concentration dependent thermodynamic stability was studied by urea equilibrium transition curves followed by fluorescence and far-UV CD spectroscopy. Kinetic unfolding and refolding experiments, including rapid double and triple mixing techniques, allowed to unravel the complex folding behavior of N-BAR. The equilibrium unfolding transition curve can be described by a two-state process, while the folding kinetics show four refolding phases, an additional burst reaction and two unfolding phases. All fast refolding phases show a rollover in the chevron plot but only one of these phases depends on the protein concentration reporting the dimerization step. Secondary structure formation occurs during the three fast refolding phases. The slowest phase can be assigned to a proline isomerization. All kinetic experiments were also followed by fluorescence anisotropy detection to verify the assignment of the dimerization step to the respective folding phase. Based on these experiments we propose for N-BAR two parallel folding pathways towards the homodimeric native state depending on the proline conformation in the unfolded state.

## Introduction

Understanding of the folding mechanism of proteins is a key challenge in molecular biophysics. The classical view of protein folding elucidates folding pathways derived from the detection and analysis of populated intermediates in equilibrium or kinetic studies[1,2]. In the recent decades the majority of studies focused on monomeric proteins gaining detailed insights into the principles of the protein folding process[3,4,5,6,7]. However, dimeric and multisubunit proteins are often of biological significance[8]. During folding of dimeric proteins specific intermolecular interaction extend the folding process. This association of the monomers can happen at different times during formation of the native state: (i) Folding and association happen simultaneously, mostly for small dimeric proteins, which fold after a two-state model where only unfolded monomers and native dimers are detectable. The Arc repressor was one of the first dimeric proteins studied in detail following a two-state folding model[9]. Other homodimeric proteins such as ORF56 or hPyA1 also show no detectable folding intermediates and are well described by a two-state folding model[10,11]. (ii) Alternatively, the contact area of each monomer must fold first before dimerization can happen and an intermediate state in the folding pathway becomes detectable. For example the DNA binding domain of the human papillomavirus protein E2 as well as the yeast triosephosphate isomerase show the building of such a monomeric intermediate before the native dimer is formed[12,13]. (iii) The unfolded monomers first built a dimeric intermediate state before the native dimer is formed. FIS and H2A/H2B show such a fast association of the monomers near the diffusion limit followed by a slower folding step to build the native dimer[14,15]. These issues have additionally to be taken into account compared to monomeric proteins. Despite this additional level of complexity many dimeric proteins show two-state transitions in equilibrium and kinetic studies. The folding of other dimeric proteins could not be described by pure two-state or three-state transitions[16,17]. Mallam *et al*. found for example by a detailed folding analysis of YibK from *Heamophilus influenzae* parallel pathways of two different intermediates which fold via a third monomeric intermediate to the final native dimer[18]. Dimeric antibody domains also show complex folding mechanisms with many observable folding events. The association of the monomers at different folding stages can be limited for example by a proline *cis*/*trans* isomerization[19]. For the antibody domain C_H_3 from MAK33 it was reported that the dimerization can only occur for chains with the native proline isomer[20].

**Fig 1:**
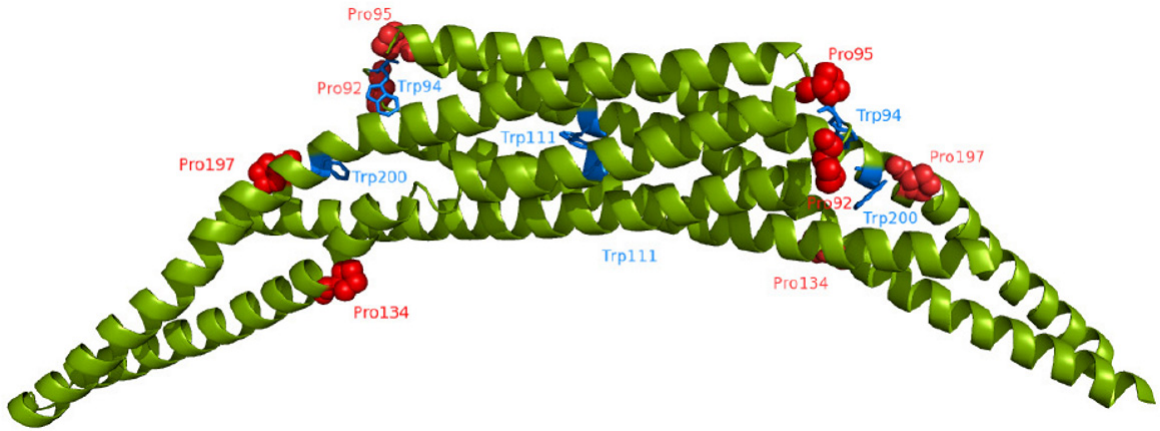
Structure of the human AmphiphysinII/Bin1 N-BAR domain. Ribbon representation of the quaternary structure of homodimeric N-BAR (PDB code: 2FIC). The three tryptophan residues per monomer are labeled in blue, the four proline residues per monomer in red spheres. The representation was prepared using PyMol.

The AmphiphysinII/Bin1 N-BAR domain belongs to the BAR (Bin, Amphiphysin, RVS167) domain superfamily and their members were identified as important regulators in eukaryotic membrane remodelling processes[21,22]. They are important components in building clathrin-coated pits and the biogenesis of T-tubules in muscles[23,24]. The structure of all archetypal BAR domains (Fig 1) shows an all-α helical homodimer with a banana shaped curvature to deform membranes[21,25]. The monomers are long kinked coiled-coil α helices which form a six-helix bundle parallel to the dimer interface. The helix kinks are also highly conserved and are located between the six helix bundle and the arm region[25,26]. In most structurally know BAR domains prolines are located at these positions. The association of the monomers defines the radius of curvature[27]. The concave surface of the dimer exhibits clusters of positively charged amino acids, which specifically bind to lipids with negatively charged headgroups[21,28].

BAR domains with an amphipathic helix (helix0) at their N-terminus are called N-BAR and exhibit a higher activity during tubulation. These helices are only stable in the presence of lipids, otherwise they are unstructured[29]. This could be investigated by different biophysical methods including paramagnetic resonance spectroscopy (EPR), nuclear magnetic resonance (NMR) and circular dichroism (CD)[29,30,31]. The amphiphatic character of helix0 is important because changing the hydrophobic side to a more polar surface reduces membrane binding and tubulation[32]. Two main mechanisms are discussed how N-BAR bends lipid bilayer: the scaffolding mechanism and the hydrophobic insertion mechanism[30,33].

Here, we provide a detailed study on the folding mechanism of the homodimeric N-BAR domain by combining various biophysical methods including intrinsic tryptophan fluorescence, fluorescence anisotropy and CD spectroscopy. Urea equilibrium unfolding transitions show no intermediates, whereas kinetic single- and double-mixing experiments suggest a folding mechanism with two parallel pathways via monomeric and dimeric intermediates with different *cis*/*trans* prolyl conformations and structural properties. The analyses of fluorescence anisotropy and far-UV CD measurements underline the complex folding behavior.

## Materials and Methods

### Protein expression and purification

The AmphiphysinII/Bin1 N-BAR domain plasmid was a kind gift of E. D. Laue (Cambridge)[25]. The plasmid was transformed in *E*.*coli* BL21(DE3) cells. Overexpression was induced with 1mM IPTG at OD_600_ 0.7 before growing for four hours at 37°C. After harvesting the cell pellet was resuspended in 50 mM Na phosphate, 300 mM NaCl, 20 mM imidazol, pH 8.0, containing protease inhibitor (Sigma Aldrich P2714).

Cells were lysed by lysozyme for 30 min and subsequently treated in a microfluidic fluidizer. The lysate was clarified by centrifugation for one hour. The supernatant was loaded on a nickel Sepharose column, washed with resuspension buffer and eluted with a gradient up to 350 mM imidazol. N-BAR was detected using SDS-polyacrylamid gel electrophoresis and stained with Coomasie blue. Fractions containing N-BAR were pooled. The His_6_-tag was cleaved by thrombin and simultaneous dialyzed against 20 mM Tris/HCl, 150 mM NaCl at pH 8.0 over night to remove imidazol before a second nickel Sepharose column to separate cleaved and non-cleaved N-BAR. The flow through was loaded on a size exclusion (S75) column which was equilibrate with 20 mM Na phosphate, 100 mM NaCl pH 7.4. N-BAR containing fractions were concentrated and stored at -20°C.

### Equilibrium fluorescence and CD spectroscopy

All experiments were performed in 20 mM Na phosphate, 100 mM NaCl at pH 7.4 and 15°C. Solutions containing urea were freshly prepared to reduce the effect from reactive cyanate ions[34]. The concentration for the stock solution and every sample were determined according to Warren and Gordon[35].

Fluorescence spectra were recorded at a Jasco FP-6500 fluorescence spectrometer with an excitation wavelength of 280 nm, an emission wavelength of 310–420 nm at various protein concentrations from 0.1 μM to 25 μM. Far-UV CD spectra were recorded at a Jasco J-810 spectropolarimeter, the band pass was set to 1 nm. The change in the α-helical CD spectrum was observed in a 0.1 cm or 1 cm cuvette at 222 nm depending on the protein concentration. All equilibrium-transitions were analyzed according to a two state model for dimeric proteins 2U ↔ N_2_ [36]:

Fluorescence and CD data were fitted individually to:

$$f_{\text{u}}=\frac{Y_{\text{obs}}-Y_{\text{N}}}{Y_{\text{U}}-Y_{\text{N}}}$$ (1)

where *Y*_obs_ is the spectroscopic signal at a given urea concentration, *Y*_N_ and *Y*_U_ are the spectroscopic signals for native and unfolded protein at the same urea concentration. The fraction of unfolded monomers is defined by:

$$f_{\text{U}}=\frac{\sqrt{K_{\text{U}}^{2}+8\left[P_{0}\right]K_{\text{U}}}-K_{\text{U}}}{4\left[P_{0}\right]}$$ (2)

with [*P*_0_] representing the total monomer protein concentration and *K*_U_ by:

$$K_{\text{U}}=\text{exp}\left(-\frac{\Delta G^{0}-m\cdot \left[\text{urea}\right]}{RT}\right).$$ (3)

The transition midpoint was calculated as follows:

$$\left[Urea\right]{\;}_{0.5}=\frac{\Delta G^{0}+RT\;\text{ln}\left[P_{0}\right]}{m}$$ (4)

### Single- and double-mixing kinetics

Fast refolding and unfolding kinetics were measured with an Applied Photophysics SX-20 stopped-flow spectrometer at 15°C. The intrinsic fluorescence was observed above 320 nm using a cut-off filter after excitation at 280 nm. Unfolding was initiated after 11-fold dilution of native protein solved in 20 mM Na phosphate, 100 mM NaCl pH 7.4 with urea (>2.2 M) in the same buffer. For refolding experiments N-BAR was unfolded in 4 M urea for 30 min at 15°C before refolding was started by 11-fold dilution. All experiments were repeated five to eight times under identical conditions, analyzed and averaged using the program Origin 7.5. Unfolding reactions were fitted with first-order reactions with the required number of exponentials:

$$Y(t)=Y_{0}+\sum_{i=1}^{N}Y_{\text{i}}\cdot \text{exp}(-k_{\text{i}}t)$$ (5)

*Y*(*t*) is the signal at time *t*, *Y*_0_ is the offset, *Y*_i_ is the amplitude of the corresponding kinetic phase and *k*_i_ is the first-order rate constant of this phase. A sum of first-order reactions and one second-order reaction was fitted to refolding kinetics:

$$Y(t)=Y_{0}+Y_{1}\frac{k_{\text{app}}t}{1+k_{\text{app}}t}+\sum_{i=2}^{N}Y_{\text{i}}\;\text{exp}(-k_{\text{i}}t)$$ (6)

where *k*_app_ is the observed apparent rate constant related to the second-order rate constant *k*_2nd_ as follows:

$$k_{\text{app}}=[P_{0}]k_{2\text{nd}}$$ (7)

Fitting of Eq 6 to the fluorescence detected single-mixing refolding kinetics at low urea concentrations required *N* = 4 and to reduce the number of parameters, *k*_2_ was fixed to the value from the corresponding fluorescence anisotropy experiment and *k*_4_ to the value from manual mixing. For unfolding measurements the final protein concentration was 1 μM, for refolding measurements various concentrations from 0.1 μM to 5 μM were used. The slowest refolding reaction was measured by manual mixing and analyzed according to one first-order reaction.

Double mixing experiments were achieved for the N assay and for the U assay[37] in the same buffer conditions as described above. The N assay started from various protein concentrations between 6 μM and 30 μM N-BAR in 4 M urea (equilibrated for 30 min) followed by a 6-fold dilution with refolding buffer (1 μM to 5 μM N-BAR in 0.66 M urea) to permit refolding from 0.1 s to 300 s before 1:1 mixing with 8.3 M urea to obtain 0.5 μM to 2.5 μM N-BAR in 4.5 M urea. The achieved kinetics was analyzed with Eq 5 (*N* = 2) as described above. The obtained amplitude plot of the unfolding phases as a function of refolding time was used as an indication for the formation of intermediates and the native state. The amplitude traces of the fast unfolding phase were fitted with Eq 6 (*N* = 1) or Eq 5 (*N* = 2) for the amplitudes of the slow unfolding phase.

The U assay was initiated by unfolding of 12 μM native N-BAR in a 1:1 mixing step with 9 M urea to get 6 μM protein in 4.5 M urea. Refolding was started after various unfolding times (0.1 s to 300 s) by 6-fold dilution with refolding buffer resulting in 1 μM N-BAR in a final concentration of 0.75 M urea. The kinetic traces were analyzed using Eq 6 (*N* = 2). The obtained amplitude plots of the refolding phases as a function of unfolding time were used as an indication of the formation of unfolded protein. The observed traces were fitted with Eq 5 (*N* = 1) for the amplitudes of λ_1_, and Eq 5 (*N* = 2) for the amplitudes of λ_2_ and λ_3_.

Far-UV CD stopped-flow was observed at an Applied Photophysics PiStar CD stopped-flow spectrometer. Changes in the ellipticity were followed at 225 nm in a 1cm curvette. Refolding and unfolding kinetics were measured at urea concentrations as described for the fluorescence stopped-flow experiments. The protein concentration was 1μM after mixing for urea dependent measurements and 0.25 μM to 4 μM N-BAR for protein concentration dependent refolding experiments. Fitting of the single- and double-mixing experiments were obtained with Eqs 5 and 6 according to the described analysis of the fluorescence but with reduced *N* if phases were missing.

Kinetic fluorescence anisotropy measurements were performed with an Applied Photophysics SX-20 stopped-flow spectrometer with a T-format dual channel detector system for simultaneous detection of parallel and perpendicular emission. For all fluorescence anisotropy measurements the excitation wavelength was set to 297 nm using a xenon-mercury lamp. The intrinsic fluorescence was again followed above 320 nm using a cut-off filter. The experiments were designed and analyzed in analogy to the fluorescence measurements described above with reduced *N* in Eqs 5 and 6 if phases were missing.

## Results

### Thermodynamic stability of N-BAR

**Fig 2:**
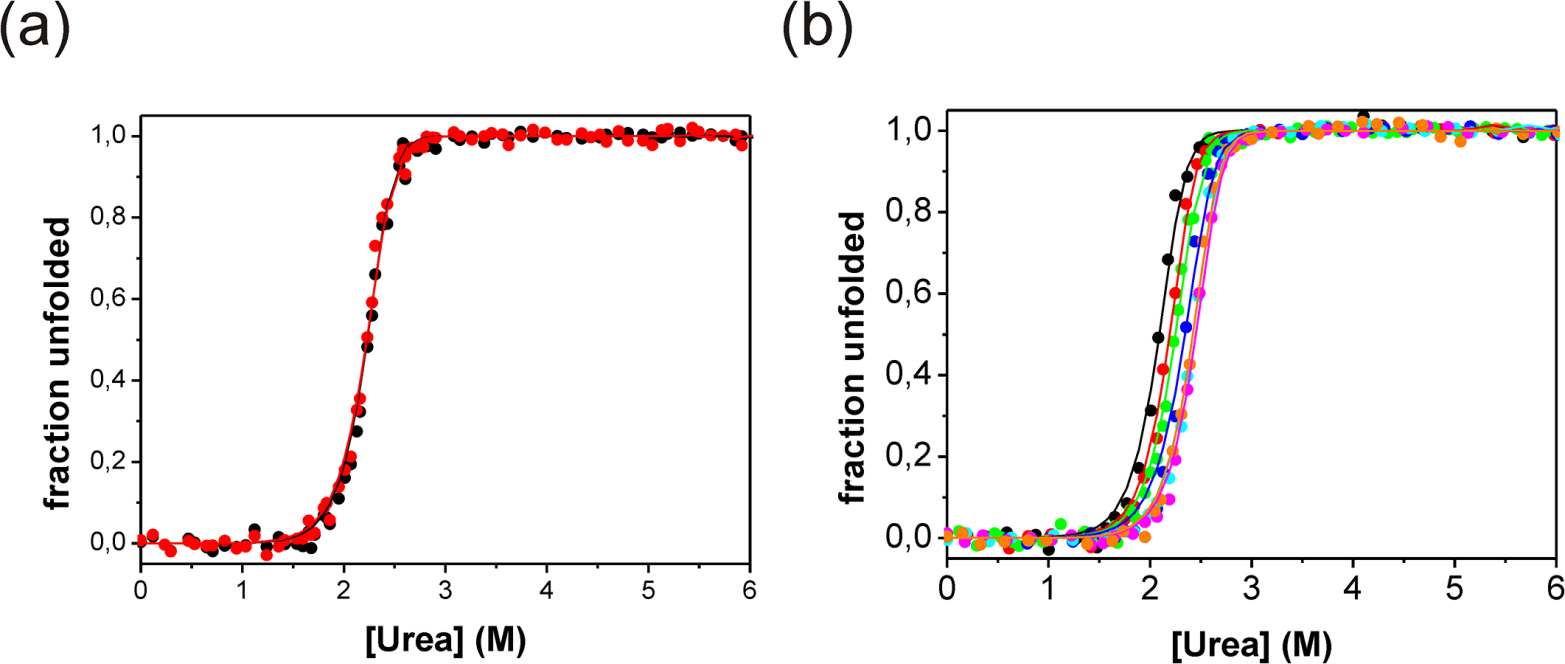
Urea induced equilibrium unfolding transitions of N-BAR. (a) The black symbols represented fluorescence (327 nm) and red symbols circular dichroismn (222 nm) measurements. The transition curves were measured with 1 μM protein in 20 mM Na phosphate, 100 mM Na chloride, pH 7.4 at 15°C. All curves were fitted to a two-state model for dimeric proteins. (b) Fluorescence detected urea transition curves of N-BAR at different protein concentrations: black circle 0.1 μM, red circle 0.5 μM, green circle 1 μM, dark blue circle 2.5 μM, light blue circle 5 μM, magenta circle 10 μM and orange circle 25 μM. The results of all fits are summarized in Table 1

The thermodynamic stability of N-BAR was investigated using its intrinsic spectroscopic properties including the change in fluorescence of the three tryptophan residues per monomer and the strong negative ellipticity of the far-UV CD spectrum (S1 File). Urea induced equilibrium unfolding curves show a single transition curve for both probes, suggesting a two-state transition at equilibrium without detectable intermediate (Fig 2A). The same curve was achieved during refolding from 7 M urea, illustrating that unfolding and refolding are completely reversible (S2 File). The received data could be analyzed by a two-state model for dimeric proteins 2U ↔ N_2_. The Gibbs free energy for unfolding (Δ*G*_u_°) was 86.1 kJ/mol with a cooperativity factor (*m*-value) of 24.3 kJ/mol^-1^M^-1^, the transition midpoint is at 2.25 M urea at 1 μM N-BAR (Table 1). A protein variant without the helix0 (Δ(1–32) BAR) has the same stability as the wildtype protein (S3 File) showing that helix0 has no significant influence on the thermodynamic stability of N-BAR in aqueous solution. Furthermore, the presence of Na sulphate has a strong stabilizing effect on the unfolding transition curves. Using 100 mM Na_2_SO_4_ the Gibbs free energy is increased by 8 kJ/mol, the transitions midpoint shift to 2.42 M while the *m*-value is constant (S4 File). The transition midpoint is also a function of the used protein concentration as expected from 2U ↔ N_2_. With increasing concentrations of N-BAR (up to 250fold) the transition midpoint shifts to higher urea concentrations (Fig 2B). The transition midpoint shifts from 2.1 M to 2.42 M. The *m*-value is about constant for all used protein concentrations (Table 1).

**Table 1:** Thermodynamic analysis of urea induced equilibrium unfolding of N-BAR.

N-BAR variant	method	protein concentration (μM)	Δ*G*_U_° (kJ/mol)	*m*–value (kJ/mol·M)	[Urea]_0,5_ (M)
wt	fluorescence	0.1	85.6±0.1	23.2±0.1	2.10±0.01
	fluorescence	0.5	85.7±0.1	24.1±0.1	2.18±0.01
	far-UV CD	0.5	85.6±0.1	23.9±0.1	2.19±0.01
	fluorescence	1.0	86.5 ±2.1	24.5±0.9	2.25±0.1
	far-UV CD	1.0	85.5±0.8	24.3±0.3	2.23±0.05
	fluorescence	2.5	86.3±0.1	24.3±0.1	2.35±0.01
	far-UV CD	2.5	85.9±0.1	24.2±0.1	2.33±0.01
	fluorescence	5	85.8±0.1	24.0±0.1	2.42±0.01
	far-UV CD	5	85.3±0.1	24.0±0.1	2.41±0.01
	fluorescence	10	84.5±0.2	24.0±0.1	2.45±0.02
	far-UV CD	10	84.9±0.1	24.1±0.1	2.43±0.01
	fluorescence	25	84.0±0.1	24.8±0.1	2.42±0.01
	fluorescence^a^	1.0	82.3±0.1	22.9±0.1	2.22±0.01
	far-UV CD^a^	1.0	81.6±0.1	22.7±0.1	2.2±0.01
ΔBAR	fluorescence	1.0	84.5 ±0.7	24.2±0.1	2.19±0.03
	far-UV CD	1.0	85.2±0.6	24.4±0.1	2.20±0.02
wt	fluorescence^b^	1.0	93.8±0.1	25.7±0.1	2.42±0.01
	far-UV CD^b^	1.0	92.8±0.8	25.7±0.3	2.39±0.05

The thermodynamic parameters were calculated with Eq 2, the indicated errors are the standard deviation from the fit.

^a^ Data were collected after dilution from 7 M urea

^b^ Data were collected with 100mM Na_2_SO_4_

### Folding kinetics of N-BAR

**Fig 3:**
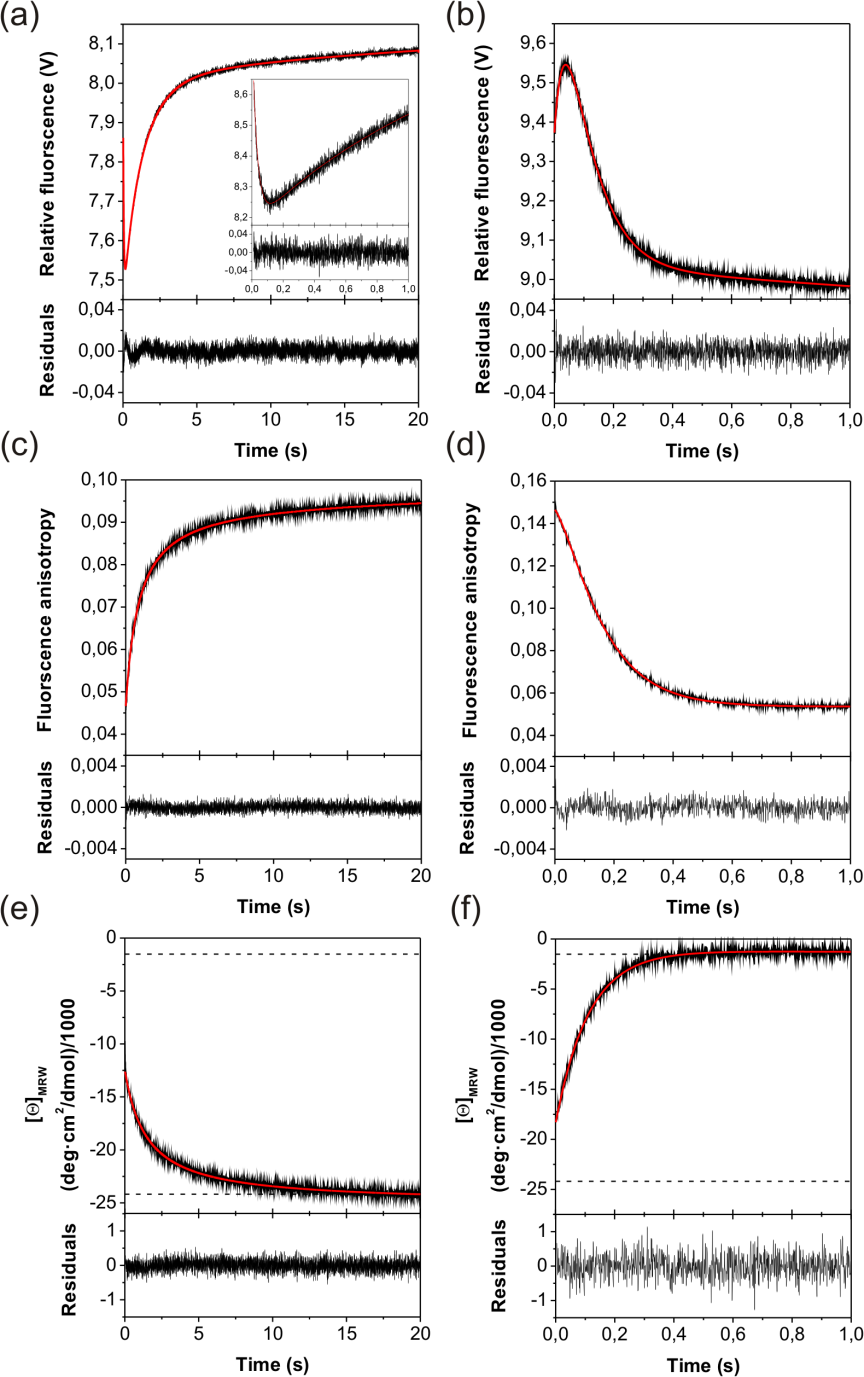
Single-mixing refolding and unfolding kinetics of N-BAR. The kinetics were monitored (a) and (b) by fluorescence, (c) and (d) by fluorescence anisotropy, (e) and (f) by far-UV CD. Refolding (a, c, e) was initiated by rapid dilution of unfolded protein in 4 M urea to 0.4 M urea. Unfolding (b, d, f) was initiated by rapid mixing of native protein in 5.5 M urea. The kinetic traces can be best described by (a) a triple-exponential and one second order function, (b) a double-exponential function, (c) and (e) one second order reaction and (d) and (f) two and one mono-exponential function, respectively. The residuals shown below each kinetic trace give the deviations of the fit from the experimental data. The insert in figure (a) shows the first second of refolding which was separately measured to improve time resolution. In (a) the rate constant of one of the three exponential functions was fixed to 0.01 s^-1^ (value from manual mixing) and one to 1.0 s^-1^ (value from fluorescence anisotropy kinetics) for fitting.

Different stopped-flow experiments were employed to investigate the kinetics of the urea-induced unfolding and refolding process. In single-mixing experiments native N-BAR was rapidly unfolded in urea above 2.3 M or refolded from 4 M to urea concentrations below 2.4 M (Fig 3). Fluorescence detected unfolding kinetics can be described by a double-exponential equation above 4.4 M urea and a single-exponential equation between 2.4 and 4.4 M urea, whereas the refolding kinetics show four different phases. The observed refolding phases are labeled (from the fastest to the slowest phases) with λ_1–4_ and the unfolding phases with λ_5–6_.

Refolding kinetics show an undershoot time course with at least one intermediate state exhibiting a lower fluorescence than unfolded and native N-BAR (Fig 3A). The increasing fluorescence trace shows within the same time window two different processes but only one phase (λ_2_) depends on the protein concentration (S5 File). For a final urea concentration at 0.4 M urea three refolding reactions were observed after stopped-flow mixing with rate constants of λ_1_ = 25 s^-1^, λ_2_ = 6.2 · 10^5^ M^-1^·s^-1^, and λ_3_ = 0.6 s^-1^. Only λ_2_ depends on the used protein concentration and thus monitors the dimerization reaction of two monomers. Additionally a fourth refolding phase was observed after long term unfolding of N-BAR in 4 M urea. This reaction was measured separately by manual mixing and showed a rate constant of λ_4_ = 0.01 s^-1^.

The unfolding curve can be described as a biphasic process under strong unfolding conditions with rate constants of λ_5_ = 24.2 s^-1^ and λ_6_ = 7.5 s^-1^ at 5.5 M urea (Fig 3B). The observed overshoot kinetics indicates a transiently populated intermediate during unfolding with a higher fluorescence compared to native and unfolded N-BAR. Unfolding kinetics below 4.4 M urea shows only a single unfolding phase (λ_6_). At the corresponding urea concentration λ_5_ is only visible in interrupted refolding experiments. Below 3 M urea λ_6_ was measured with manual mixing (S6 File).

**Fig 4:**
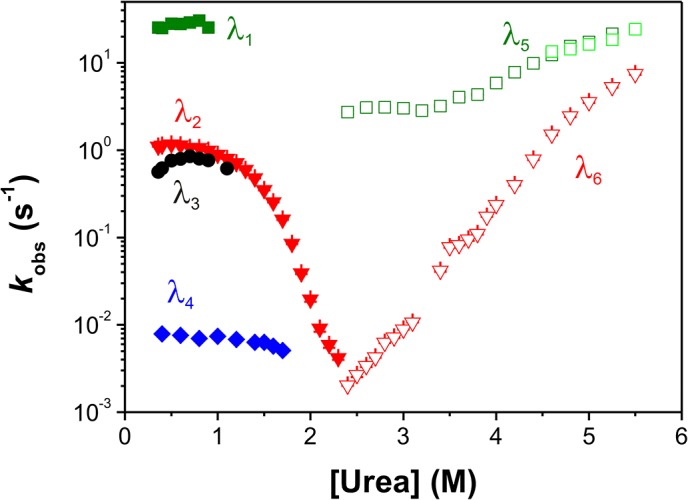
Chevron plot for the fluorescence detected un- and refolding kinetics. Urea-dependence of the apparent refolding and unfolding rate constants of N-BAR measured by fluorescence: open symbols represent unfolding kinetics, green squares show the fast unfolding phase measured with double mixing experiments, light green squares show the fast unfolding phase measured with single-mixing experiment above 4.4 M urea, red triangles show the slow unfolding phase measured with single-mixing stopped-flow and below 3.5 M urea by manual mixing experiments. Closed symbols represent refolding kinetics, green squares, red triangles and black circles show single-mixing stopped-flow kinetics. Blue diamonds symbolize very slow kinetics from manual mixing experiments.

Additionally to these single-mixing refolding and unfolding experiments, time resolved fluorescence anisotropy was measured (Fig 3C and 3D), because the anisotropic tumbling of the different folding states of N-BAR should change during the formation and disappearance of the elongated structure of the homodimer. Such detected unfolding kinetics can be best described by two single-exponential functions with the same rate constants as observed in fluorescence kinetics described above. The respective refolding experiment exhibited single phase kinetics depending on the protein concentration. Consequently, this reaction is a second order reaction representing the dimerization step. Because of this property and the value of the refolding rate constant, this refolding phase corresponds to λ_2_ observed in the fluorescence kinetics. To reduce the number of free parameters during kinetic analysis of the latter fluorescence kinetics (Fig 3A and the chevron-plot in Fig 4), we fixed λ_2_ to the value observed by fluorescence anisotropy.

The chevron-plot for the fluorescence detected kinetics contains further details (Fig 4). The fast unfolding rate constant (λ_5_) shows a weak urea dependence and increases only about 10 fold between 2.5 M and 5.5 M urea. In the same range the slow unfolding rate constant (λ_6_) increases by a factor of 10000. Both unfolding phases measured by fluorescence anisotropy show the same dependence in the chevron-plot (data not shown) as measured by fluorescence. The refolding phases could be determined between 0.4 M and 2.3 M urea. Below 1 M urea four refolding phases could be resolved. The rate constants for λ_1_ and λ_4_ are almost independent from the urea concentration. The slowest refolding process (λ_4_) is assumed to result from a peptidyl-prolyl *cis*/*trans* isomerization reaction[38,39]. It should be mentioned that all proline residues are in the *trans* conformation in the native state according to the crystal structure of N-BAR. We tried to accelerate this very slow reaction with peptidyl-prolyl isomerases, a specific class of enzymes which catalyses this types of reactions. However, in the presence of different isomerases no significant acceleration of this reaction could be observed. λ_2_ shows the strongest urea dependence of all refolding events; this suggests major conformational rearrangements related to this phase. Between 0.4 M and 1 M urea this phase shows a rollover. With fluorescence anisotropy measurements we could verify this phase and thus the analysis of the single-mixing experiments. The third refolding phase λ_3_ is only observable between 0.4 M to 1.2 M urea and shows even a slight acceleration up to 0.7 M urea.

**Fig 5:**
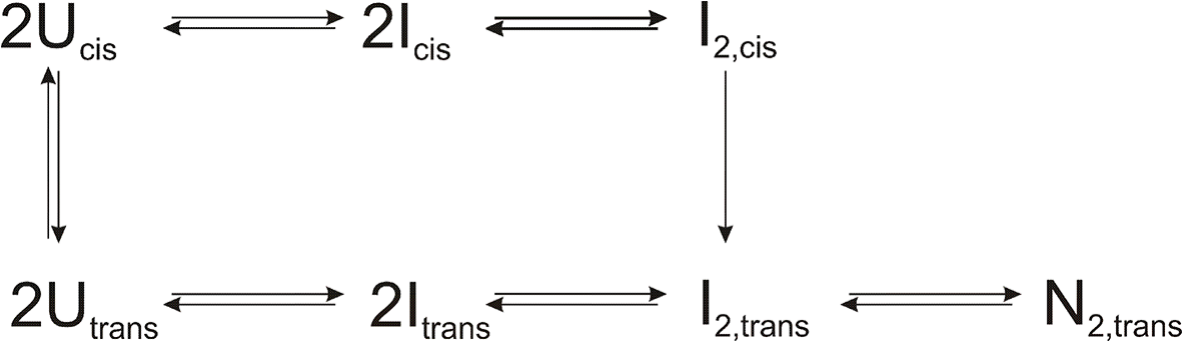
Proposed folding mechanism of N-BAR.

From these results and experiments discussed below we suggest a folding mechanism for N-BAR presented in Fig 5. In the unfolded protein there is an equilibrium between prolyl *cis* and *trans* conformations resulting in two parallel pathways[40]. The first two steps of both folding pathways are independent of the prolyl conformation: very rapid formation of a monomeric intermediate I followed by a slower dimerization to I_2_. At this state the dimeric intermediates proceed differently: intermediates with the native *trans* prolyl conformation (I_2,trans_) can directly fold to the native form. Intermediates with the corresponding *cis* prolyl conformation (I_2,cis_) first isomerize very slowly into the native prolyl isomer before forming the dimeric native state N_2,trans_. To confirm this proposed folding mechanism, the now following experiments have been performed.

### Double-mixing experiments verify the N-BAR folding mechanism

**Fig 6:**
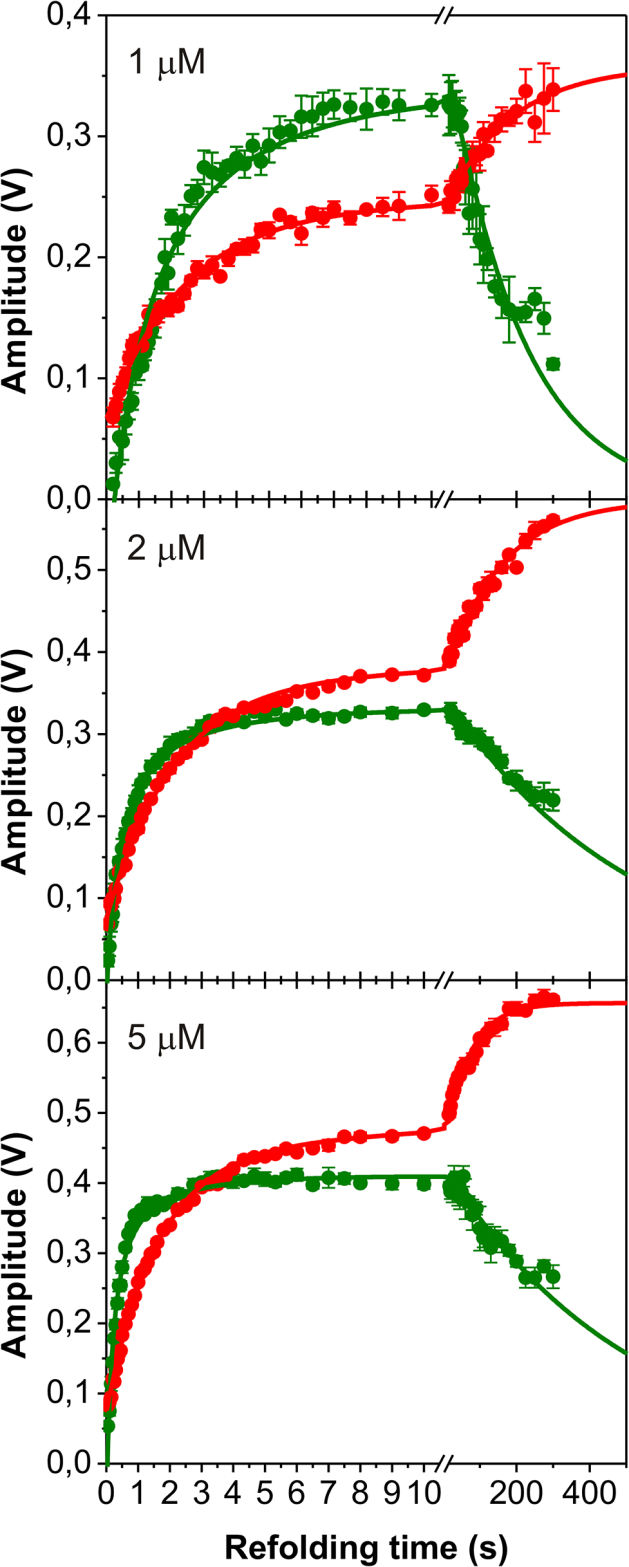
Double mixing refolding kinetics of N-BAR (N assay). The amplitude plot of the double mixing experiment reveals the time course of population of native and intermediate species during refolding. Amplitudes of the fast unfolding reaction are labeled by green symbols and amplitudes of the slow unfolding reaction are depicted in red. The final protein concentration is indicated in the plot. Continues lines represent a fit of a double-exponential function for the red line and a second order reaction plus one exponential function for the green line. The results of the fit are summarized in Table 2.

To assign the various un- and refolding phases to the respective intermediate states and their order of appearance we performed different double-mixing stopped-flow experiments[38,41,42,43]. The first set of experiments comprises the so called N assay to quantify the build up of native and intermediate states during refolding. Here unfolded N-BAR was first diluted in 0.66 M urea to initiate refolding and after different time intervals refolding was interrupted by dilution to 4.5 M urea where native N-BAR unfolds slowly while partly folding states unfold more rapidly. Under these conditions two unfolding processes with opposite sign of amplitudes occur with rate constants of λ_5_ = 6.4 s^-1^ and λ_6_ = 0.5 s^-1^. The same two phases are observed in single-mixing experiments at this urea concentration (see Fig 4). The amplitude values of both reactions change with the proceeding refolding process (Fig 6) and thus disclose parts of the folding mechanism of N-BAR. The green filled symbols in Fig 6 correspond to the amplitudes of the fast unfolding process (λ_5_) and show the build up of an intermediate state with a maximum at about 10s before it slowly decays again. The red filled symbols indicate the slow unfolding process (λ_6_) and its amplitudes show the generation of the final native state. Thus the progression of amplitudes of both unfolding phases reports about three different refolding steps of N-BAR. To determine during which phase the dimerization reaction happens we repeat this N assay at three different protein concentrations, because the association of monomers should be concentration dependent. Only the amplitudes of the fast unfolding process (green in Fig 6) were significantly concentration dependent. All observed rate constants at the respective protein concentration are summarized in Table 2.

**Table 2:** Summary of refolding rate constants obtained from the double-mixing N assay.

Concentration of N-BAR during refolding	Amplitudes from the fast unfolding phase (λ_5_)		Amplitudes from the slow unfolding phase (λ_6_)	
	λ_2_ in M^-1^·s^-1^	λ_4_ in s^-1^	λ_3_ in s^-1^	λ_4_ in s^-1^
1 μM	2.6·10^5^±2.4·10^4^	0,012±0,001	0.42±0,02	0.006±0,001
2 μM	4.8·10^5^±2.4·10^4^	0,005±7·10^−4^	0.48±0,02	0.006±0,001
5 μM	4.3·10^5^±3.5·10^4^	0.006±0,001	0.63±0,02	0.015±0,001

Together, the N assay revealed the following features: First, the fastest refolding reaction with a rate constant λ_1_ = 25 s^-1^ observed in single-mixing experiments is not present here. This implies that this refolding reaction occurs before the rate-limiting steps of the formation of N_2,trans_. Second, both amplitude plots show biphasic behavior during refolding. The amplitudes for λ_5_ first increase with about 4·10^5^ s^-1^·M^-1^ before they decrease with a rate of 0.01 s^-1^ towards lower amplitudes. The amplitudes monitored by λ_6_ show a biphasic increase with two different rate constants of 0.69 s^-1^ and 0.01 s^-1^ showing that this reaction produces native protein N_2,trans_. All three observed phases were also observed in the single-mixing refolding experiments at this urea concentration (see Fig 4). Third, only the increasing amplitudes from λ_5_ depend on the protein concentration and thus show the formation of a dimeric intermediate I_2_. The subsequent formation of N_2,trans_ happens with a protein concentration independent rate constant of 0.69 s^-1^ (red circles in Fig 6) corresponding to λ_3_ and represent the formation of native N-BAR. There is no observable lag phase in the formation of native protein as would be expected for a sequential pathway (see below). Fourth, a very slow refolding reaction (0.01 s^-1^) towards the native protein leads to an increase of the amplitudes of λ_6_ and a decrease of the amplitudes of λ_5_. This shows that the formation of one fraction of native molecules is significantly retarded. Because of the low rate constant, proline isomerization most probably is the reason for this process which limits for this fraction the formation of native dimers. The opposite sign of the amplitudes of this very slow folding step (red vs. green symbols in Fig 6) suggest a sequential folding process here.

**Fig 7:**
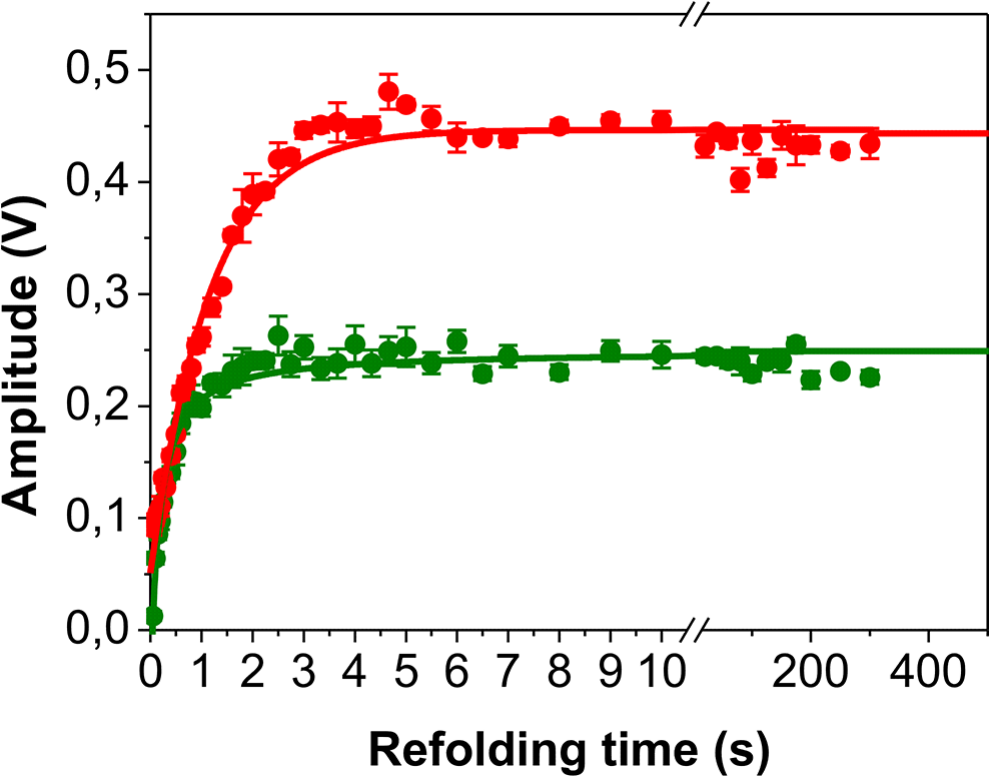
Triple mixing refolding kinetics of N-BAR. The amplitude plot of the triple-jump experiment was monitored by changes in fluorescence. The unfolding to 4 M urea was accomplished manually with a delay time of 15 s (±3 s) followed by a double mixing refolding experiments (N assay). The protein concentration during the refolding time was 2 μM. Amplitudes from the fast unfolding reaction are labeled with green symbols; amplitudes from the slow unfolding reaction are labeled with red symbols. The fits (same functions as for the N assay but without the very slow exponential decay) are represented by continues lines.

To further unravel the connection between the various folding steps of N-BAR we perform a triple jump experiment[44,45]. Here the first mixing step was performed manually with a delay time of approximately 15s (±3s) to unfold N-BAR in 4 M urea following the N assay which was described above. The observed unfolding kinetics also showed two-phase kinetics with the same rate constantans as mention for the N assay. The results of this experiment are shown in Fig 7. The observed refolding rates derived from the time course of amplitudes during the first 10s of refolding are the same for the N assay and the triple jump experiment. In contrast, for extended refolding times the observed amplitudes remain constant for the triple jump. This results from the short unfolding time of only 15 s before refolding started, which prevents prolyl isomerization in the unfolded state and all proline residues are in the native *trans* conformation when refolding starts. The relative amplitudes of both phases also differ from the previous double mixing experiment. After 10s of refolding the relative amplitudes have the same ratio in the triple jump experiment compared to the double mixing experiment after 300s. The relative loss in the amplitudes of the fast unfolding reaction in the first 10s of refolding in the triple jump experiment (green symbols in Fig 7) also underline that I_2,trans_ is formed but no intermediate states with non-native prolyl isomers accumulate in this time window. This shows that complete refolding occurs in the first 10s in the triple jump experiment because all unfolded molecules contain the prolyl residue in its native conformation and the upper pathway in Fig 5 gets not populated.

We also repeated the N assay with fluorescence anisotropy and far-UV CD (see below) detected stopped-flow experiments (Fig A in S8 File and S9 File). Interestingly, these double-mixing experiments showed biphasic unfolding kinetics behavior for both probes. For both amplitude plots we observed the same time course and the same rate constants of refolding as measured by fluorescence. Therefore, significant changes in the secondary structure and global shape occur during the transitions between the monomeric, dimeric and native states.

**Fig 8:**
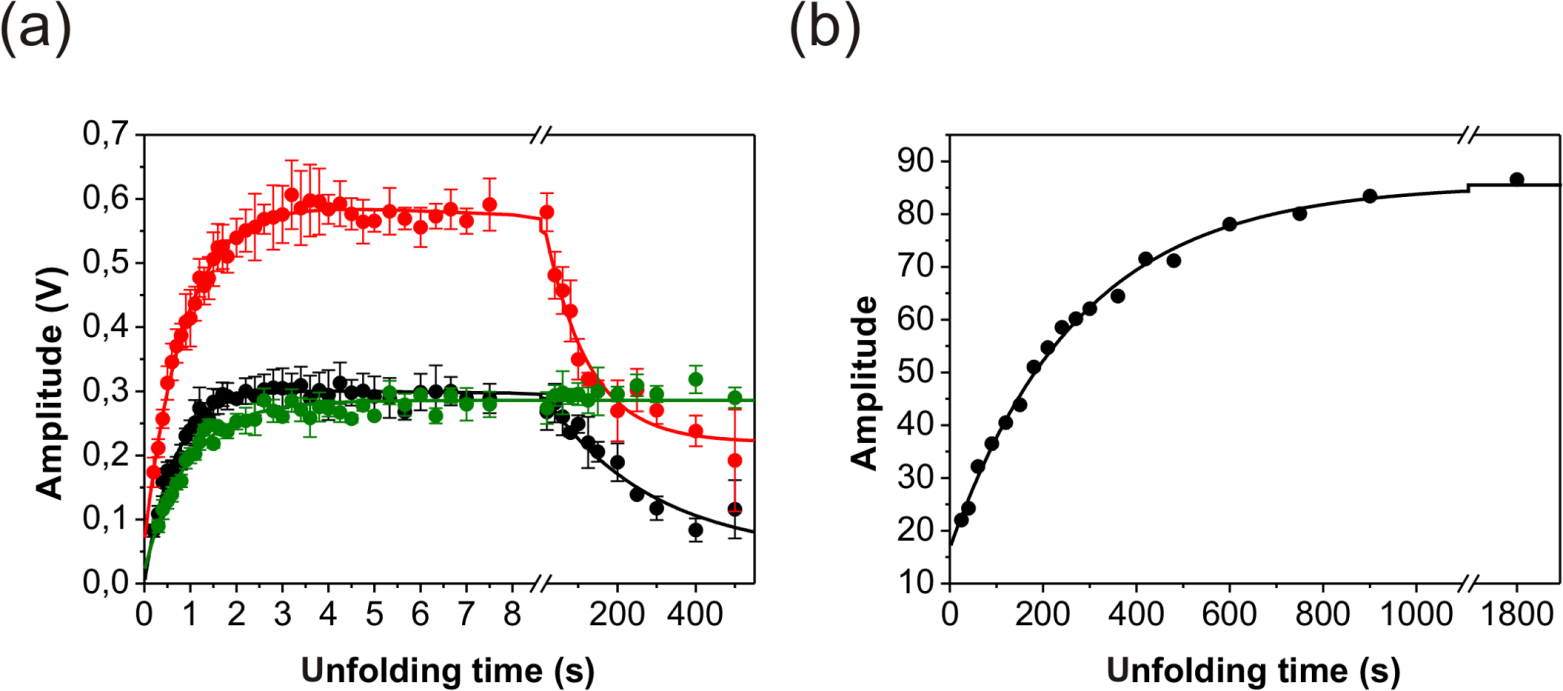
Double mixing unfolding kinetics of N-BAR (U assay). The amplitude plot for the fluorescence detected double mixing experiments reports about the time course of state during unfolding of N-BAR. (a) The colors of the three amplitude traces correspond to the colors of λ_1_ (green), λ_2_ (red), and λ_3_ (black) in Fig 4. The protein concentration was 1 μM finally. The continuous lines correspond to a single-exponential (green line) or double-exponential fit (red and black line) of the respective refolding phase. (b) Amplitude plot of the slowest refolding phase (λ_4_) against the unfolding time (manual mixing).

In the U assay first native N-BAR was unfolded in 4.5 M urea for increasing time intervals before refolding was initiated by dilution to 0.75 M urea and the kinetic trace was observed by fluorescence. Under these conditions three refolding phases were observed with the same rate constants as observed in the single-mixing refolding experiments at 0.75 M urea. The corresponding amplitudes of the refolding phases depend on the unfolding time (Fig 8A), which contains some information about the intermediates formed during unfolding. The green filled symbols in Fig 8A correspond to the amplitudes of the fast refolding phase λ_1_ and report about the formation of the fully unfolded protein U. The black filled symbols represent the amplitudes of the second order reaction (λ_2_) and the red filled symbols of the slow refolding reaction (λ_3_). For short unfolding times (< 8 s) all unfolded chains fold back to the native state within 20 s because no *cis/trans* isomerization could happen in the unfolded state. All three amplitudes from the refolding phases show the same time course during this unfolding time window. Their calculated single-exponential rate constants are for the amplitudes from the fast refolding phase 1.06 s^-1^, for the middle phase 1.15 s^-1^ and for the slow phase 1.54 s^-1^. These rate constants agree within error well with the slowest unfolding phase λ_6_ at 4.5 M urea and show that this unfolding phase is the initial and rate-limiting unfolding step. For unfolding times longer than 8 s isomerization in U becomes significant and influences the amplitudes. The slowest refolding phase (λ_4_) becomes observable (Fig 8B) and shows a single-exponential increase with a rate constant of 0.0036 s^-1^. Therefore, this rate corresponds to the *trans* → *cis* isomerization in the unfolded state. The amplitudes from the fastest refolding phase λ_1_ are insensitive to this isomerization (green symbols between 8 s and 500 s in Fig 8A). The amplitudes from λ_2_ and λ_3_ decrease with the rate constant of this isomerization. For λ_3_ this is obvious because the I_2,trans_ to N_2,trans_ transition occurs after the rate limiting I_2,cis_ to I_2,trans_ step for molecules refolding from U_*cis*_. For the decrease of λ_2_ amplitudes with unfolding time we can only speculate that this might result from the concentration dependence of this phase when the number of U_*trans*_ molecules decrease and of U_*cis*_ increase.

Finally, we performed the U assay under fluorescence anisotropy detection. Here only one refolding phase (λ_2_) is observed and the amplitude of this reaction increases single-exponentially with unfolding time and a rate constant of 1.8 s^-1^ (Fig B in S8 File). This rate constant agrees well with the above mentioned λ_6_ detected in single- and double-mixing experiments.

### Secondary structure changes during folding

To investigate, during which of the so far described phases major changes of the secondary structure occur, we measured refolding and unfolding kinetics by stopped-flow far-UV CD. Interestingly, both refolding and unfolding kinetics contained only one observable phase (Fig 3E and 3F). Refolding kinetics depends again on the protein concentration and corresponds to λ_2_. Additionally, a burst phase is observable in far-UV CD stopped-flow, showing α-helical structure formation in the dead time of the experiment. The ratios of the start- and end-values did not depend on the protein concentration (S7 File), suggesting that dimer association does not occur during the burst phase. The slowest refolding phase (λ_4_) is also not visible after long term unfolding and therefore has no major secondary structure changes are expected for this phase.

**Fig 9:**
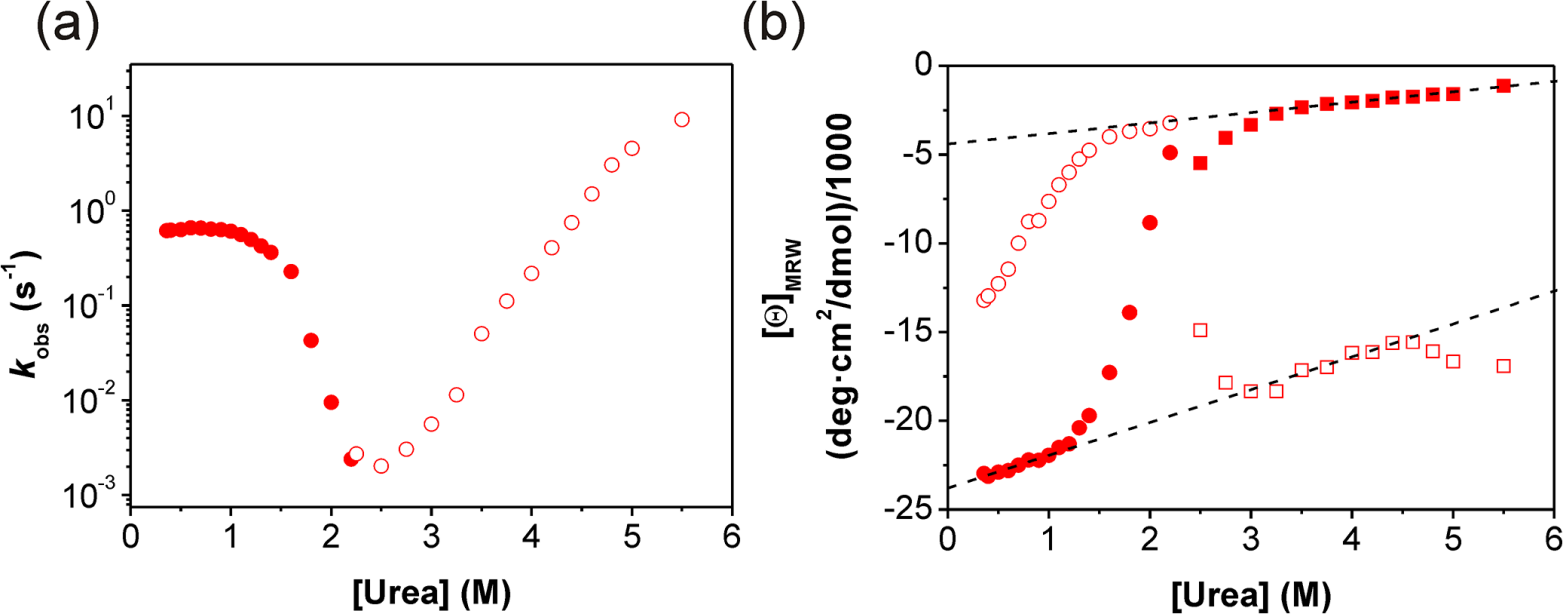
Chevron plot and end point analysis for the far-UV CD detected un- and refolding kinetics. Un- and refolding kinetics of N-BAR measured by far-UV circular dichroismn as a function of the urea concentration. (a) Chevron-plot of the apparent rate constants. Filled symbols represent refolding kinetics while open symbols stand for unfolding kinetics. (b) Analysis of the endpoints from refolding (circles) and unfolding measurements (squares). Closed symbols represent the final signal and open symbols the initial signal.

CD detected unfolding and refolding kinetics were monophasic under all measured conditions between 0.4 M urea and 5.5 M urea (Fig 9A). The apparent rate constants for both reactions coincide in the transition region very well because of the microscopic reversibility of the unfolding and refolding reaction. The urea dependence of the unfolding reaction varies linearly (in the semi-logarithm plot) over a broad range of the urea concentration and shows a slight curvature above 5 M urea matching the fluorescence detected chevron plot at this urea concentration (Fig 4). This might indicate an intermediate, a weak movement of the rate-limiting transition state or non-linear denaturant activity. The refolding reaction shows a strong deviation from linearity in the low urea concentration ranges (rollover effect) and indicates very fast formation of an intermediate which cannot be resolve in our experiment[46,47]. The urea dependence of the detected phases agrees very well with λ_2_ (refolding) and λ_5_ (unfolding) detected by fluorescence changes. Confirmation of a burst phase event is given by the amplitude of the folding kinetics, where CD signals were plotted against the corresponding urea concentration (Fig 9B). The end-values show a two-state transition curve close to the measured urea-induced equilibrium unfolding curve (Fig 2). The start-values for the refolding reaction clearly deviate from a linear urea dependence at low urea concentrations showing a rapid formation of secondary structure during the burst phase[4].

## Discussion

In the present study we investigated the equilibrium and kinetics of urea induced un- and refolding of the AmphiphysinII/Bin1 N-BAR domain by fluorescence, fluorescence anisotropy and far-UV CD spectroscopy. Because of the dimeric character of N-BAR the apparent thermodynamic stability monitored by equilibrium unfolding transition curves depends on the protein concentration. It was not possible to detect an equilibrium folding intermediate under equilibrium conditions. The calculated thermodynamic stability with 85.6 kJ/mol is in the range of 42 to 120 kJ/mol found for other dimeric proteins[48].

The kinetic folding studies demonstrate the complexity of N-BAR folding with four detectable refolding phases and two detectable unfolding phases revealing various transient intermediates with spectroscopic properties differing from the unfolded and fully folded state. With fluorescence spectroscopy we can distinguish between changes in the local environment during folding (fluorescence) and the fixation of the chromophore towards the non-isotropically tumbling framework of the forming N-BAR (fluorescence anisotropy). Our proposed folding (Fig 5) represents a minimal model for the folding mechanism with a fast-refolding and a slow-refolding pathway resulting from a heterogeneous unfolded state of N-BAR. It cannot be ruled out, that further folding steps exist which are not observable by fluorescence techniques or far-UV CD stopped-flow.

The simple two-state model which was observed in equilibrium unfolding curves cannot sufficiently explain our kinetic measurements because such a model predicts on single-exponential kinetics and a V-shaped chevron plot independent from the observable[49]. In far-UV CD single-mixing stopped-flow experiments we found only one refolding and unfolding phase covering the complete change in ellipticity at 225 nm. The observed refolding rate constant depends on the protein concentration and thus corresponds to the dimerization of N-BAR. The refolding phase shows a rollover towards low urea concentration in the chevron-plot. This behavior is indicative for an intermediate state[50]. We propose that this is an *on-pathway* intermediate, most probably corresponding to I_*trans/cis*_, where the formation of the intermediate is fast compared to the following rate-limiting dimerization reaction, which has a higher energy barrier than the formation of the intermediate. The presence of an *on-pathway* intermediate can be best verified by a lag-phase for the following reaction (λ_2_) in a double-mixing experiments, if the consecutive steps have about the same time constant. We did not observe a lag-phase for the built up of the dimeric state probably because formation of this state is more than 20fold slower compared to the formation of the monomeric intermediate. The existence of an intermediate is also underlined by the start-end value analysis which shows a loss in the amplitudes at low urea concentrations and an early accumulation of the intermediate in the inaccessible time window of a stopped-flow experiments[51,52]. I_*trans/cis*_ is of monomeric character because the ratio of the start and end values does not change with increasing protein concentrations[14,15]. Formation of I_*trans/cis*_ via λ_1_ could be directly detected only by the more sensitive fluorescence probe. Thus, with these far-UV CD experiments we can only see one part of our complete folding mechanism. We address the dimerization reaction to the main refolding event because it is observable with every probe used in this study.

The measured fluorescence kinetics show the full complexity of N-BAR folding and the following properties: (i) a heterogeneous mixture of unfolded chains concerning *trans*- and *cis*-prolyl peptide bonds which determines the time course of quaternary structure formation, (ii) the proline isomerization does not influence the dimerization reaction and (iii) the dimerization reaction is the main refolding step regarding concomitant secondary and quaternary structure formation. Single-jump refolding kinetics using far-UV CD and fluorescence show a different number of refolding events suggesting that not all fluorescence detected refolding events are associated with secondary structure formation. The refolding phases λ_3_ and λ_4_ occur in the dimeric state which has close to native secondary structure.

The double-mixing refolding experiments monitor the same refolding events (Figs 6 and 7, S8 File and S9 File) independently of the probes (fluorescence or far-UV CD). The slowest refolding event which is proposed to be a prolyl *cis*/*trans* isomerization is not rate limiting for formation of the dimer. The fluorescence detected unfolding kinetics show single phase kinetics and only above 4.5 M urea two phase kinetics in fluorescence measurements suggesting that below 4.5 M urea the slow unfolding phase (λ_6_) becomes rate-limiting. Far-UV CD detected unfolding is even more sensitive to this rate limitation by λ_6_, because λ_5_ is not observable in single-jump experiments. The latter phase is only detectable in the N assay because I_2,trans_ gets populated via the unfolded state and not only from N_2,trans_.

That folding starts from chains with the native *trans*-conformation or with a non-native *cis*-conformation of prolyl peptide bonds in the unfolded state has been reported for other proteins[45]. Independently of the *cis*/*trans* conformation, folding starts with a very fast folding event leading to the formation of a monomeric intermediate. As mentioned we assume that the burst-phase intermediate observed in far-UV CD and the fastest observed refolding phase (λ_1_) observed under fluorescence detection monitor the same folding event. A direct far-UV CD signal change in single-mixing experiments corresponding to λ_1_ could not be resolved although the dead-time of 30 ms for this experiment is sufficient to detect a reaction with a rate constant of 25 s^-1^. The reason for this observation remains unclear.

This monomeric intermediate dimerizes independently of the prolyl conformation. The absence of a second protein concentration dependent reaction excludes a second dimerization reaction and suggests a sequential folding pathway. For other dimeric proteins including P22 Arc repressor, knotted protein YibK, and T4 fibritin, a protein concentration dependent process could also be observed and assign to the dimerization step[9,18,53]. The rate constant of the dimerization of N-BAR is 6.2·10^5^ M^-1^·s^-1^at 0.4 M urea and lies three orders of magnitude below the diffusion limit of about 10^9^ M^-1^·s^-1^. This implies that the association of the two monomers is not diffusion-limited and not only a collision event but promoted by a conformational change of the monomeric intermediates. The deviation of the observed second order rate constant from linearity above 1 μM shows that dimerization is not rate-limiting under all conditions. This suggests a change in the rate-limiting step for the dimerization above this protein concentration. It should be noted that this deviation could be seen by all three probes used here. Such a behavior was also observed for other dimeric proteins such as P22 Arc repressor or ORF56[9,10].

The N assay revealed that the completely folded native dimer N_2,trans_ is not formed by the dimerization reaction but via an additional step reported by λ_3_. This reaction is not present in single-jump far-UV CD and fluorescence anisotropy refolding measurements. This implies that no substantional secondary structure rearrangements occur during this phase and that the anisotropy tumbling of the fluorophores does not change. Therefore, I_2,trans_ resembles already closely N_2,trans_ in structural terms. However, we could show that in double mixing refolding experiments under fluorescence anisotropy and far-UV CD detection the dimerization phase could be visualized with the same rate constant as in fluorescence measurements. In these N assays this reaction nearly completely disappears with the slowest refolding reaction λ_4_. This also underlines that this reaction is not an *off-pathway* intermediate and depends on the preceding dimerization reactions.

Extrapolation of the two fitted curve in the N assay to zero shows that the amplitudes from the fast unfolding phase are close to zero. The amplitudes from the slow unfolding reaction show that about 10% of native molecules are present at the beginning of refolding independent of the used protein concentration (Figs 6 and 7). This suggests that this fraction of native N-BAR can form in the dead time of the N-assay. The nature of this refolding pathway is still open. The rollover in the chevron plots and the burst in the far-UV CD measurements show that faster events occur. Whether this very fast population of N results from a direct pathway toward the native state or via a very fast forming intermediate state remains to be resolved. The N assay under fluorescence anisotropy and far-UV CD detection does not show this dead-time event (Fig A in S8 File and S9 File).

In contrast to the two faster refolding phases, λ_3_ also shows a slight acceleration with increasing urea concentration, which is typically the signature of an unfolding process. In other proteins this effect was interpreted as indication for the formation of an *off-pathway* intermediate[4,54]. Because λ_3_ is not protein concentration dependent we can exclude that a non-productive dimer or higher oligomer gets formed. If an *off-pathway* intermediate is formed after dimerization the unfolding of this intermediate would be rate-limiting in the formation of the native state with λ_3_ and thus be pronounced in a lag-phase of the λ_6_ amplitudes in the U assay, which we do not observe. Therefore, the urea dependence of λ_3_ remains unclear.

We assigned the slowest refolding reaction to a peptidyl-prolyl *cis/trans* isomerisation. Although this phase could not be accelerated with an isomerase, this phase has a rate-constante of 0.01 s^-1^, nearly no urea dependence in the chevron-plot and an activation energy of 85 kJ/mol which is characteristic for a peptidyl-prolyl *cis/trans* isomerisation[40]. Each N-BAR monomer contains four proline residues which are all in the *trans*-conformation in the native state. In principle, all of the Xaa-Pro peptide bonds can produce slow isomerization reactions because in the unfolded state an equilibrium between the *cis* and *trans* conformation can evolve[40,55]. The ratio between both conformations is determined by the preceding amino acid. There exists one Trp94-Pro95 peptide bound which is proposed to have a high *cis* fraction in the unfolded state in comparison to other amino acids[56,57]. For peptides with a preceding Trp it was shown that the *cis* content is up to 34%. The isomerization of this peptide bound leads to a local change in the chemical environment of the preceding tryptophan and thus to a change in the fluorescence. Along these lines, the isomerization of Trp94-Pro95 is most probably the main reason for the observable slow refolding phase λ_4_.

A further hint that the slowest refolding reaction results from a peptidyl-prolyl *cis/trans* isomerization comes from the triple-jump experiment, which prepares only U_*trans*_ before the N assay. Here no further change in the amplitudes is observable with refolding times longer than 10 s. Additionally, the amplitudes from λ_5_ which report the dimerization reaction do not reach the high plateau value observed in the N assay started from a *cis/trans* equilibrium. This result underlines that without isomerization in the unfolded state no refolding retardation by λ_4_ takes place and thus the entire upper folding pathway in Fig 5 starting from U_*cis*_ is missing.

In our proposed model we assign both dimeric states–the intermediate and native state–in all-*cis* or in all-*trans*. In principle mixed dimeric states could form. λ_4_ does not depend on the protein concentration and λ_3_ and λ_4_ give no change in the far-UV CD spectrum. Therefore, I_2,cis_ has to be a dimer and both, I_2,cis_ and I_2,trans_ have native secondary structures. The latter might also prevent PPIases to reach the prolyl peptide bond for catalysis. This implies that the major structural rearrangements during refolding can occur independent from the prolyl conformation and thus mixed dimers (one *cis* and one *trans* prolyl monomer unit) should be possible. A clear answer about their appearance or absence can not be given by the presented experiments.

Unfolding follows a sequential pathway with N_2,trans_ → I_2,trans_ as rate-limiting unfolding step. This unfolding phase is detectable with all three employed spectroscopic probes corresponding to λ_6_. The following unfolding phase λ_5_ is best pronounced in the fluorescence experiments under strong unfolding condition. Below 4.5 M urea this phase is only detectable by double-mixing experiments, which populate I_2,trans_ via refolding and thus becoming detectable again by all three spectroscopic probes. Rate limiting λ_6_ is also the reason that both performed U assays (fluorescence and fluorescence anisotropy detected) only contained the slowest unfolding phase. Such a behavior was also observed for other proteins[58] for the same reason. A third unfolding phase (I_trans/cis_ → U_trans/cis_), which is expected from our folding model, is not visible in any experiment. We assume a very fast kinetics and/or probably a small amplitude for this final unfolding step.

In terms of the biological function of N-BAR we could show by the equilibrium studies that the N-terminal helix0 does not influence the thermodynamic stability of N-BAR, at least in the absence of membrane lipids. The radius of curvature of N-BAR is defined by kinks in the helix bundle at well conserved positions giving rise to the dimensions of membrane tubules [27]. The here revealed folding kinetics of N-BAR are also determined by proline residues. Whether folding retardation and function are related by the conserved prolines as found for other proteins [59] e.g. by systematic alanine substitution, is future work.

## Supporting Information

S1 File Equilibrium spectra of nativ and unfolded N-BAR. Spectra were detected with fluorescence (**Fig A**) and far-UV CD spectroscopy (**Fig B**). The black line shows the nativ protein while the unfolded protein in 7 M urea is represented by the red line. All data were obtained at 1 μM protein in 20 mM Na phosphat, 100 mM Na chlorid, pH 7.4 and 15°C as described in Materials and Methods.
(PDF)

S2 File Reversibility test for urea induced un- and refolding. Urea transition curve of N-BAR detected by fluorescence (**Fig A**) and far-UV CD (**Fig B**). Black symbols indicate the transition curve started from native protein and red symbols show the curve from unfolded protein in 7 M urea.
(PDF)

S3 File Urea induced equilibrium transition curves of ΔBAR. Urea induced unfolding curve of (1–32)BAR measured with fluorescence (black circles) and circular dichroismn (red circles) (**Fig A**). The two curves show a two-state transition and superimpose very well. Comparison of the transition curves of N-BAR (black circles) and BAR (red circles) measured with fluorescence (**Fig B**).
(PDF)

S4 File Urea induced equilibrium transition curves of N-BAR. Transition curves of N-BAR measured by fluorescence in the absence (black circles) and presence (red circles) of 100 mM Na_2_SO_4_.
(PDF)

S5 File Plot of the apparent second order rate constant of N-BAR refolding at 0.4M urea under fluorescence detection (corresponding to Fig 3A) as a function of protein concentration. (PDF)

S6 File Fluorescence detected unfolding trace of N-BAR after manual mixing. Unfolding was initiated by manual mixing in 3 M urea, 20 mM Na phosphat, 100 mM Na chlorid, pH 7.4 and 15°C at a protein concentration of 1 μM. The kinetic trace was detected at 327 nm and can be best described by a single-exponential function.
(PDF)

S7 File Plot of the ratio of initial and final CD value as a function of N-BAR concentration. (PDF)

S8 File Double mixing refolding kinetics of N-BAR detected by fluorescence anisotropy. Amplitude plot of the N assay measured with fluorescence anisotropy (**Fig A**). Green symbols represent the fast unfolding phase while red symbols represent the amplitudes from the slow unfolding phase. The fitted rate constants from red amplitudes are for both protein concentrations λ_3_ = 0.5 (±0.05) s^-1^ and λ_4_ = 0.01 (± 5·10^−3^) s^-1^ which are comparable to single-mixing and double mixing fluorescence experiments. The second order rate constant from the green symbols differ slightly from fluorescence measurements. The rate constant of the amplitude decay is the same. Amplitude plot of the fluorescence anisotropy U assay (**Fig B**). The only observable rate constant is λ_6_ = 1.75 s^-1^ which is the same rate constant observed in single-mixing experiments measured with all three probs.
(PDF)

S9 File Double mixing refolding kinetics of N-BAR (N assay) detected by far-UV CD-spectroscopy. Amplitude plot of the far-UV detected N assay. Green circles show the amplitudes from the fast unfolding phase and red circles from the slow unfolding reaction. The calculated rate constants are in the same range as measured in fluorescence.
(PDF)
