# Supplementary material for: Protein Folding Mechanism of the Dimeric AmphiphysinII/Bin1 N-BAR Domain
Source: PLoS One. 2015 Sep 14;10(9):e0136922. doi: 10.1371/journal.pone.0136922 (PMC4569573; doi:10.1371/journal.pone.0136922)
Supplement: S1 File — Spectra were detected with fluorescence (Fig A) and far-UV CD spectroscopy (Fig B). The black line shows the nativ protein while the unfolded protein in 7 M urea is represented by the red line. All data were obtained at 1 μM protein in 20 mM Na phosphat, 100 mM Na chlorid, pH 7.4 and 15°C as described in Materials and Methods. (PDF) [file pone.0136922.s001.pdf]

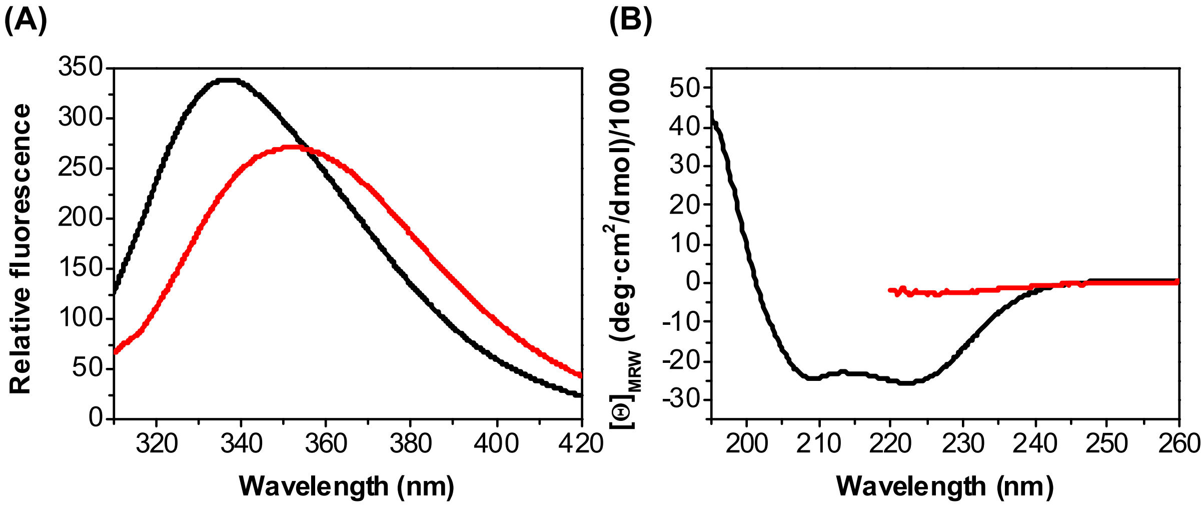

S1 File. Equilibrium spectra of nativ and unfolded N-BAR. Spectra were detected with fluorescence (**Figure A**) and far-UV CD spectroscopy (**Figure B**). The black line shows the nativ protein while the unfolded protein in 7 M urea is represented by the red line. All data were obtained at 1 µM protein in 20 mM Na phosphat, 100 mM Na chlorid, pH 7.4 and 15°C as described in Materials and Methods.
