# Supplementary material for: Protein Folding Mechanism of the Dimeric AmphiphysinII/Bin1 N-BAR Domain
Source: PLoS One. 2015 Sep 14;10(9):e0136922. doi: 10.1371/journal.pone.0136922 (PMC4569573; doi:10.1371/journal.pone.0136922)
Supplement: S2 File — Urea transition curve of N-BAR detected by fluorescence (Fig A) and far-UV CD (Fig B). Black symbols indicate the transition curve started from native protein and red symbols show the curve from unfolded protein in 7 M urea. (PDF) [file pone.0136922.s002.pdf]

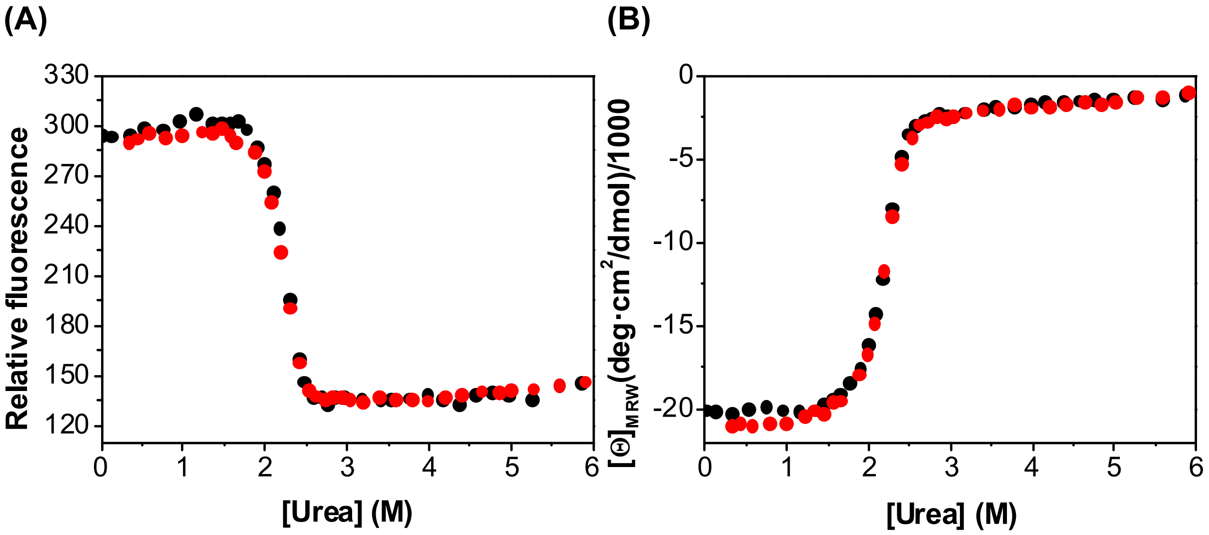

S2 File. Reversibility test for urea induced un- and refolding. Urea transition curve of N-BAR detected by fluorescence (**Figure A**) and far-UV CD (**Figure B**). Black symbols indicate the transition curve started from native protein and red symbols show the curve from unfolded protein in 7 M urea.
