# Supplementary material for: Protein Folding Mechanism of the Dimeric AmphiphysinII/Bin1 N-BAR Domain
Source: PLoS One. 2015 Sep 14;10(9):e0136922. doi: 10.1371/journal.pone.0136922 (PMC4569573; doi:10.1371/journal.pone.0136922)
Supplement: S3 File — Urea induced unfolding curve of (1–32)BAR measured with fluorescence (black circles) and circular dichroismn (red circles) (Fig A). The two curves show a two-state transition and superimpose very well. Comparison of the transition curves of N-BAR (black circles) and BAR (red circles) measured with fluorescence (Fig B). (PDF) [file pone.0136922.s003.pdf]

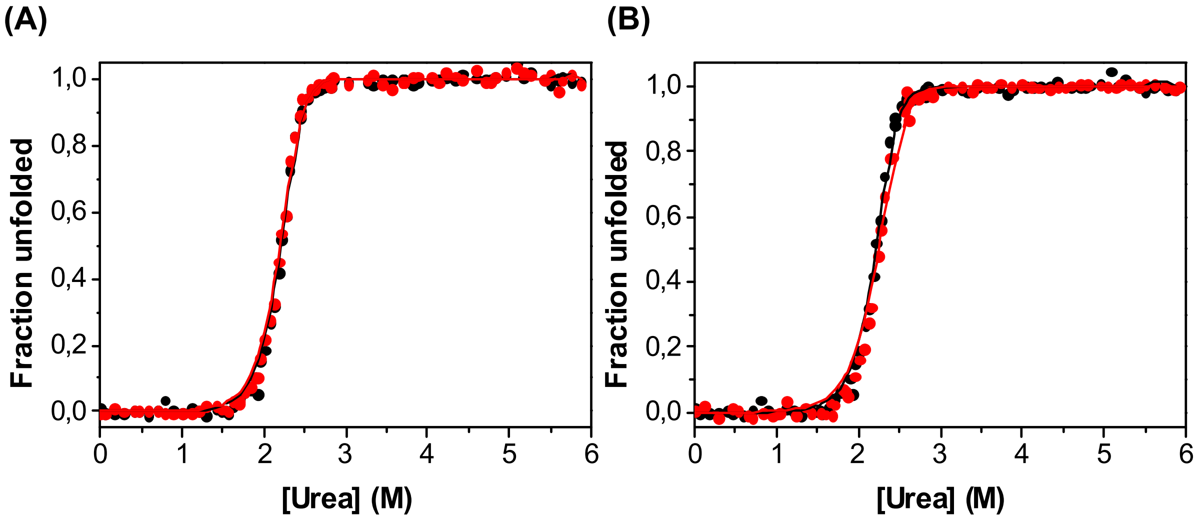

S3 File. Urea induced equilibrium transition curves of ΔBAR. Urea induced unfolding curve of Δ(1-32)BAR measured with fluorescence (black circles) and circular dichroismn (red circles) (**Figure A**). The two curves show a two-state transition and superimpose very well. Comparison of the transition curves of N-BAR (black circles) and ΔBAR (red circles) measured with fluorescence (**Figure B**).
