# Supplementary material for: Protein Folding Mechanism of the Dimeric AmphiphysinII/Bin1 N-BAR Domain
Source: PLoS One. 2015 Sep 14;10(9):e0136922. doi: 10.1371/journal.pone.0136922 (PMC4569573; doi:10.1371/journal.pone.0136922)
Supplement: S4 File — Transition curves of N-BAR measured by fluorescence in the absence (black circles) and presence (red circles) of 100 mM Na2SO4. (PDF) [file pone.0136922.s004.pdf]

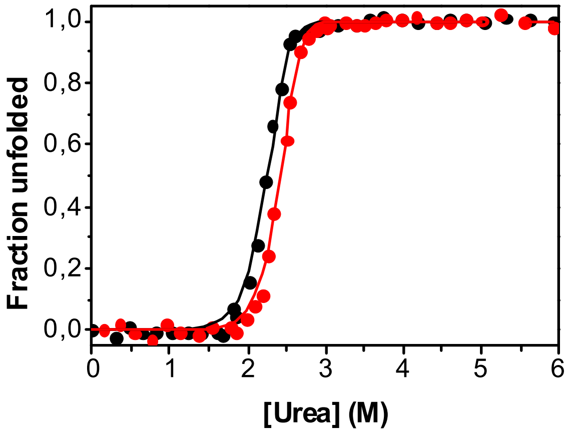

S4 File. Urea induced equilibrium transition curves of N-BAR. Transition curves of N-BAR measured by fluorescence in the absence (black circles) and presence (red circles) of 100 mM Na_2SO_4
