# Supplementary material for: Protein Folding Mechanism of the Dimeric AmphiphysinII/Bin1 N-BAR Domain
Source: PLoS One. 2015 Sep 14;10(9):e0136922. doi: 10.1371/journal.pone.0136922 (PMC4569573; doi:10.1371/journal.pone.0136922)
Supplement: S5 File — (PDF) [file pone.0136922.s005.pdf]

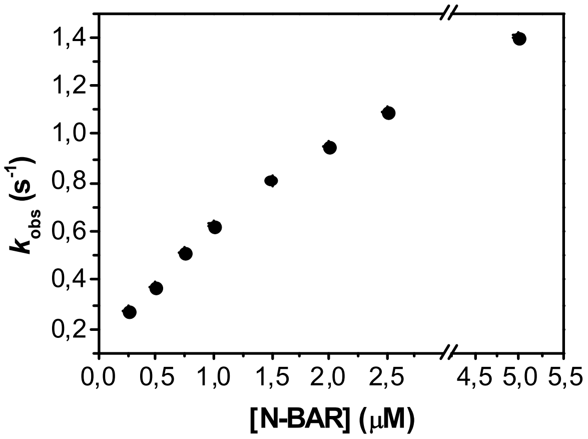

S5 File. Plot of the apparent second order rate constant of N-BAR refolding at 0.4M urea under fluorescence detection (corresponding to Fig. 3a) as a function of protein concentration.
