# Supplementary material for: Protein Folding Mechanism of the Dimeric AmphiphysinII/Bin1 N-BAR Domain
Source: PLoS One. 2015 Sep 14;10(9):e0136922. doi: 10.1371/journal.pone.0136922 (PMC4569573; doi:10.1371/journal.pone.0136922)
Supplement: S6 File — Unfolding was initiated by manual mixing in 3 M urea, 20 mM Na phosphat, 100 mM Na chlorid, pH 7.4 and 15°C at a protein concentration of 1 μM. The kinetic trace was detected at 327 nm and can be best described by a single-exponential function. (PDF) [file pone.0136922.s006.pdf]

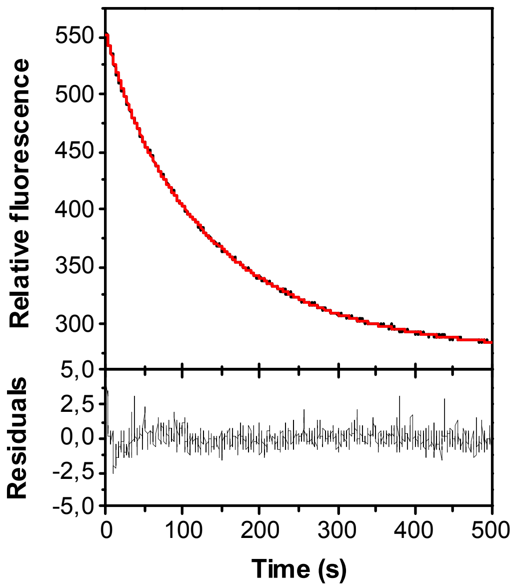

S6 File. Fluorescence detected unfolding trace of N-BAR after manual mixing. Unfolding was initiated by manual mixing in 3 M urea, 20 mM Na phosphat, 100 mM Na chlorid, pH 7.4 and 15°C at a protein concentration of 1 μM. The kinetic trace was detected at 327 nm and can be best described by a single-exponential function.
