# Supplementary figures and images for: Protein Folding Mechanism of the Dimeric AmphiphysinII/Bin1 N-BAR Domain
Source: PLoS One. 2015 Sep 14;10(9):e0136922. doi: 10.1371/journal.pone.0136922 (PMC4569573; doi:10.1371/journal.pone.0136922)

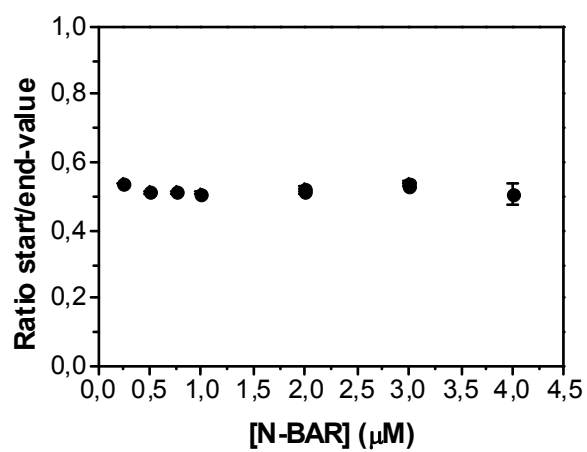

**S7 File. Plot of the ratio of initial and final CD value as a function of N-BAR concentration.**

Supplement: S7 File — (PDF) [file pone.0136922.s007.pdf]
