# Supplementary material for: Protein Folding Mechanism of the Dimeric AmphiphysinII/Bin1 N-BAR Domain
Source: PLoS One. 2015 Sep 14;10(9):e0136922. doi: 10.1371/journal.pone.0136922 (PMC4569573; doi:10.1371/journal.pone.0136922)
Supplement: S8 File — Amplitude plot of the N assay measured with fluorescence anisotropy (Fig A). Green symbols represent the fast unfolding phase while red symbols represent the amplitudes from the slow unfolding phase. The fitted rate constants from red amplitudes are for both protein concentrations λ3 = 0.5 (±0.05) s-1 and λ4 = 0.01 (± 5·10−3) s-1 which are comparable to single-mixing and double mixing fluorescence experiments. The second order rate constant from the green symbols differ slightly from fluorescence measurements. The rate constant of the amplitude decay is the same. Amplitude plot of the fluorescence anisotropy U assay (Fig B). The only observable rate constant is λ6 = 1.75 s-1 which is the same rate constant observed in single-mixing experiments measured with all three probs. (PDF) [file pone.0136922.s008.pdf]

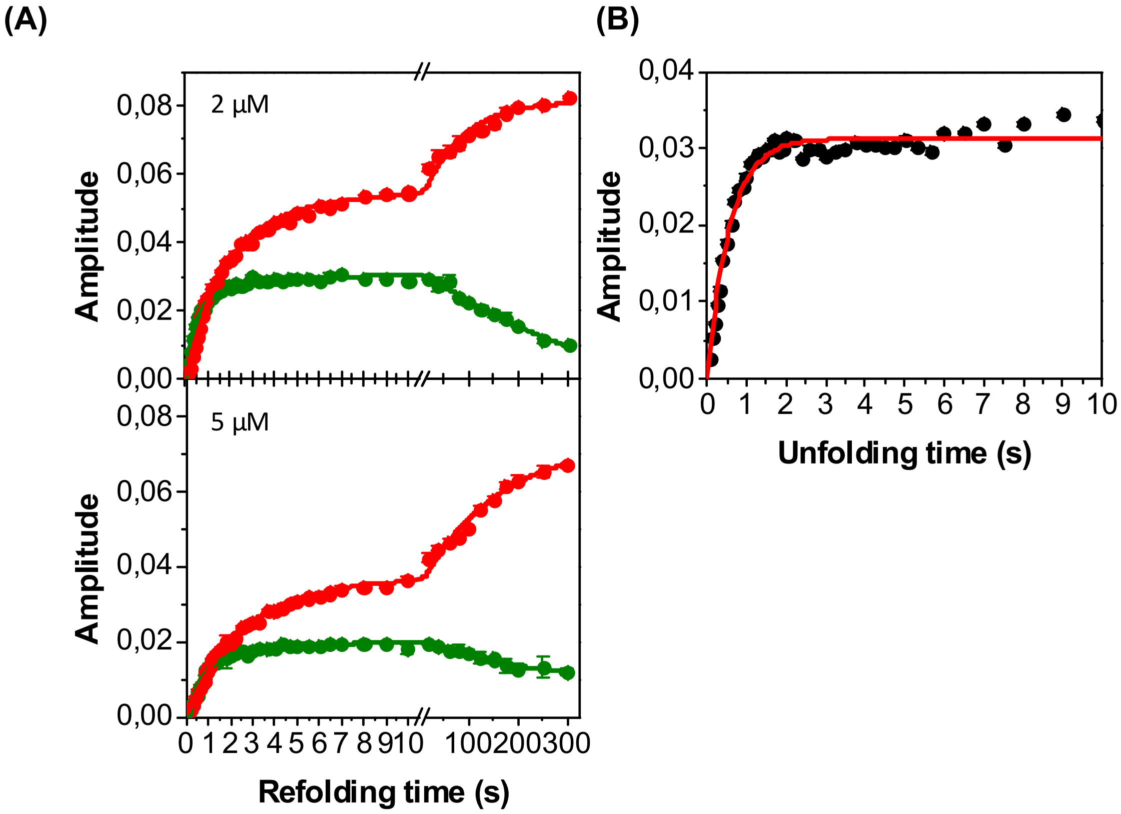

S8 File. Double mixing refolding kinetics of N-BAR detected by fluorescence anisotropy. Amplitude plot of the N assay measured with fluorescence anisotropy (**Figure A**). Green symbols represent the fast unfolding phase while red symbols represent the amplitudes from the slow unfolding phase. The fitted rate constants from red amplitudes are for both protein concentrations $\lambda_3 = 0.5\ (\pm 0.05)\ s^{-1}$ and $\lambda_4 = 0.01\ (\pm\ 5 \cdot 10^{-3})\ s^{-1}$ which are comparable to single-mixing and double mixing fluorescence experiments. The second order rate constant from the green symbols differ slightly from fluorescence measurements. The rate constant of the amplitude decay is the same. Amplitude plot of the fluorescence anisotropy U assay (**Figure B**). The only observable rate constant is $\lambda_6 = 1.75\ s^{-1}$ which is the same rate constant observed in single-mixing experiments measured with all three probs.
