# Supplementary material for: Protein Folding Mechanism of the Dimeric AmphiphysinII/Bin1 N-BAR Domain
Source: PLoS One. 2015 Sep 14;10(9):e0136922. doi: 10.1371/journal.pone.0136922 (PMC4569573; doi:10.1371/journal.pone.0136922)
Supplement: S9 File — Amplitude plot of the far-UV detected N assay. Green circles show the amplitudes from the fast unfolding phase and red circles from the slow unfolding reaction. The calculated rate constants are in the same range as measured in fluorescence. (PDF) [file pone.0136922.s009.pdf]

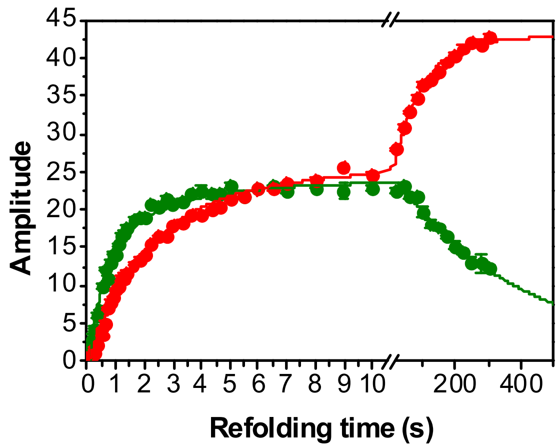

S9 File. Double mixing refolding kinetics of N-BAR (N assay) detected by far-UV CD-spectroscopy. Amplitude plot of the far-UV detected N assay. Green circles show the amplitudes from the fast unfolding phase and red circles from the slow unfolding reaction. The calculated rate constants are in the same range as measured in fluorescence.
